# Neural correlates of feedback processing in decision-making under risk

**DOI:** 10.3389/fnhum.2012.00204

**Published:** 2012-07-06

**Authors:** Beate Schuermann, Tanja Endrass, Norbert Kathmann

**Affiliations:** Department of Psychology, Humboldt-Universität zu BerlinBerlin, Germany

**Keywords:** decision-making, feedback processing, P200, P300, FRN

## Abstract

**Introduction:** Event-related brain potentials (ERPs) provide important information about the sensitivity of the brain to process varying risks. The aim of the present study was to determine how different risk levels are reflected in decision-related ERPs, namely the feedback-related negativity (FRN) and the P300. **Materials and Methods:** Twenty participants conducted a probabilistic two-choice gambling task while an electroencephalogram (EEG) was recorded. Choices were provided between a low-risk option yielding low rewards and low losses and a high-risk option yielding high rewards and high losses. While options differed in expected risks, they were equal in expected values and in feedback probabilities. **Results:** At the behavioral level, participants were generally risk-averse but modulated their risk-taking behavior according to reward history. An early positivity (P200) was enhanced on negative feedbacks in high-risk compared to low-risk choices. With regard to the FRN, there were significant amplitude differences between positive and negative feedbacks on high-risk choices, but not on low-risk choices. While the FRN on negative feedbacks did not vary with decision riskiness, reduced amplitudes were found for positive feedbacks in high-risk relative to low-risk choices. P300 amplitudes were larger in high-risk decisions, and in an additive way, after negative compared to positive feedback. **Discussion:** The present study revealed significant influences of risk and valence processing on ERPs. FRN findings suggest that the reward prediction error signal is increased after high-risk decisions. The increased P200 on negative feedback in risky decisions suggests that large negative prediction errors are already processed in the P200 time range. The later P300 amplitude is sensitive to feedback valence as well as to the risk associated with a decision. Thus, the P300 carries additional information for reward processing, mainly the enhanced motivational significance of risky decisions.

## Introduction

A significant function of the human brain is to assess the riskiness of decisions in order to prevent negative outcomes. Brain imaging studies indicate that frontolimbic brain circuits involving the ventromedial prefrontal cortex, amygdala, insula, ventral striatum, and anterior cingulate cortex (ACC), are implicated in risk processing. In particular, the ACC is important for detecting and evaluating unfavorable outcomes (Bush et al., 2000; Luu et al., 2000), and for risk assessment (Ernst et al., 2004; Fukui et al., 2005; McCoy and Platt, 2005). Greater ACC activity predicts enhanced error avoidance (Johansen and Fields, 2004; Frank et al., 2005) and less risk-taking behavior (Paulus and Frank, 2006). An influential model of decision-making under risk is the prospect theory (Tversky and Kahneman, 1981). It proposes that human decision makers are generally risk avoiding when choosing between alternatives. Nevertheless, it has been shown that risk-taking behavior may also depend on the context (Tversky and Kahneman, 1981), i.e., risk aversion increases after gains and decreases after losses.

Studies using event-related brain potentials (ERPs) have revealed that the human brain is able to evaluate the outcome of actions within a few 100 ms. Specific brain potentials are elicited by self-generated responses and performance feedback (Holroyd and Coles, 2002). The error-related negativity (ERN; Falkenstein et al., 1990; Gehring et al., 1990) and the feedback-related negativity (FRN; Miltner et al., 1997) are elicited by erroneous responses and by negative feedback or losses, respectively. ERN and FRN are assumed to originate from the anterior midcingulate cortex (Gehring and Willoughby, 2002; Debener et al., 2005). Therefore, ERN and FRN may reflect similar mechanisms of monitoring and controlling behavior. It has been suggested that the ACC uses reinforcement learning (RL) signals conveyed by the midbrain dopamine system to optimize future decision-making behavior (Holroyd and Coles, 2002). According to the RL theory, ERN and FRN reflect a reward prediction error signal in the ACC that occurs when ongoing events are worse than expected. Subsequently, the ACC triggers an adaptive modification of behavior by relating actions with their consequences (Holroyd and Coles, 2002; Rushworth et al., 2004). Another ERP component that has been shown to carry important information for reward processing is the feedback-related P300, a parietally distributed positivity (Yeung and Sanfey, 2004; Polezzi et al., 2009). It has been suggested that the feedback-related P300 may reflect the extent to which information is motivationally significant or salient (for a review, see Nieuwenhuis et al., 2005). In line with that, the P300 amplitude varies with the motivational significance of feedback information (Yeung and Sanfey, 2004; Polezzi et al., 2009) and is increased in individuals who attributed more meaning to feedback (de Bruijn et al., 2004).

Economic decision theories presume that risk depends on potential losses and increases with its probability and magnitude (Tversky and Kahneman, 1981; Brown and Braver, 2007). In this regard, rational decisions are made on the basis of the expected value, which is a multiplicative combination of the two components (Machina, 1982). Recent studies investigated the different components of risk-taking by assessing the influences of feedback valence, magnitude, and probability on ERP amplitudes. Research on the impact of the probability of feedback has generally shown that both the FRN and the P300 are modulated by this variable, with unexpected feedback being associated with enhanced amplitudes (Holroyd et al., 2004; Hajcak et al., 2007). Furthermore, it was demonstrated that amplitudes are modulated by an interaction between feedback valence and expectancy: unexpected negative feedback is associated with larger amplitudes compared to unexpected positive feedback (Frank et al., 2005; Moser and Simons, 2009). In gambling paradigms, an additional important variable associated with decision-making under risk is outcome magnitude. Yeung and Sanfey (2004) studied the effects of winning or losing large or small amounts of money on the FRN and P300 and concluded that only the P300 was affected by the amount of monetary loss, whereas the FRN was insensitive to outcome magnitude. In line with this, Toyomaki and Murohashi (2005) reported effects of magnitude on the participants' subjective assessment of losses, but no effects on FRN amplitudes (see also Sato et al., 2005; Hajcak et al., 2006). Other studies reported significant magnitude effects on the FRN (Goyer et al., 2008; Wu and Zhou, 2009). However, tasks in these studies required participants to choose from alternatives without having any information about reward magnitude. To conclude, FRN and P300 seem to reflect different aspects of risk processing in economic decision-making, valence and magnitude processing, respectively.

A limitation of most previous studies is that they did not independently control for the effects of probability, magnitude, and expected value. Some studies focusing on neural correlates of feedback processing used different expected values of choices to determine learning (van der Helden et al., 2010; Schuermann et al., 2011). Furthermore, sometimes participants were unaware of possible outcome magnitudes prior to receiving feedback, and thus could not make informed choices (Goyer et al., 2008; Wu and Zhou, 2009). Finally, for gambling tasks, choices often differed in outcome probability (Yeung and Sanfey, 2004; Cohen et al., 2007). To overcome some of these limitations, we designed a gambling task in which expected risk was independently manipulated from expected values and reward probability. Specifically, participants were requested to select between a low-risk option yielding low rewards and low losses and a high-risk option yielding high rewards and high losses. Unlike traditional RL tasks used in ERP research, participants in the present task were not required to learn outcome contingencies throughout the course of the task. In this study, expected values were equal for both options. There was also no difference in reward probabilities between the low-risk and the high-risk option. Examining risk effects also requires that probabilities involved in a decision are explicitly known (Brand et al., 2006; Brown and Braver, 2007). Therefore, in the present task participants were informed about the outcome probabilities. In sum, the present task should provide a better account to assess pure risk preference and to evaluate the influence of risk parameters on ERPs.

The aim of the present study was to determine how different risk levels are reflected in decision-related ERPs, namely the FRN and the feedback-related P300. Therefore, we developed and tested a novel two-choice gambling task allowing for the examination of risk-taking in unambiguous situations (Pilot experiment). The associated electrocortical indicators of risk-taking behavior were examined in the main experiment. Considering that expected values of high-risk and low-risk options were equal, we predicted that participants are predominantly risk-averse, namely that they are less willing to choose risky options (Tversky and Kahneman, 1981). Furthermore, we assumed that participants are more risk-averse following gains and relatively more risk-seeking following losses (Tversky and Kahneman, 1981). According to the RL theory, which states that the FRN responds to the difference between experienced and anticipated rewards, we predicted enhanced FRN amplitudes for high-risk compared to low-risk decisions. We also assumed that P300 amplitudes would be enhanced for high-risk decisions compared to low-risk ones due to an enhanced motivation of risky decisions (Nieuwenhuis et al., 2005).

## Pilot experiment

### Materials and methods

#### Participants

Fifty participants (30 women and 20 men) took part in the pilot experiment. Their mean age was 30.5 years (SD: 11.4; range: 18–50). Three of the participants were left-handed. Participants had no history of neurological or psychiatric diseases. All participants received verbal and written explanations of the purpose and procedures of the study, and gave written informed consent in accordance with the Declaration of Helsinki.

#### Task and procedure

**Figure 1:**
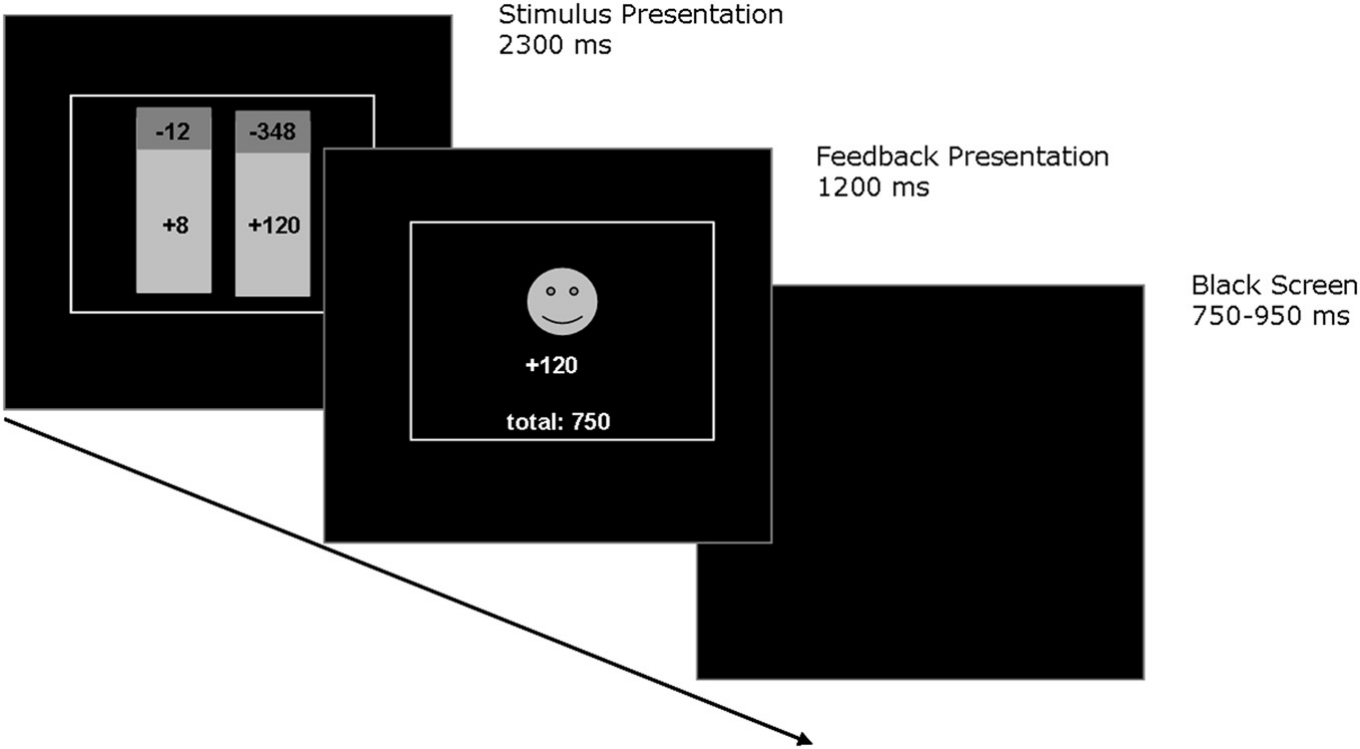
**Schematic depiction of the probabilistic two-choice gambling task.** During each trial, participants were asked to choose between two options that were represented visually by a histogram. The colors of the histogram indicated the relative probability of gaining (colored green) which was always 75% and the relative probability of losing (colored red) which was always 25%. The current amount of gains and losses associated with each option were displayed in each histogram. Choices were made by pressing one of two corresponding response buttons. After 700 ms, participants were shown the outcome associated with their choice for 1100 ms. A red frowny face together with a negative amount indicated negative feedback, while a green smiley face together with a positive amount indicated positive feedback. In addition, the current total amount was presented below the feedback stimuli.

A computerized probabilistic two-choice gambling task was administered, which involved low-risk and high-risk options. On each trial participants were asked to choose between two options that were presented on a computer screen (see Figure 1). The colors of the stimuli indicated the relative probability of winning (green), which was always 75%, and the relative probability of losing (red), which was always 25%. Reward magnitudes associated with choice options were displayed in each stimulus. Choices were made by pressing one of two corresponding response buttons. After 700 ms, participants were shown the outcome associated with the selected option for 1100 ms. A red frowny face together with a negative amount indicated negative feedback, while a green smiley face together with a positive amount indicated positive feedback. In addition, the total account balance across trials was presented below the feedback stimuli. Choices had to be made within 2300 ms, otherwise participants were prompted to respond more quickly. The next trial was presented after an intertrial interval of 750–950 ms. Following standardized written instructions, participants performed two practice trials. The pilot experiment consisted of 112 total trials and lasted about 5 min. Participants were instructed to earn as many points as possible and were told that each point corresponds to one Euro cent. Participants received on average 4.50€ in the pilot experiment. Table 1 presents an overview of the reinforcement schedule. In each trial, participants always had to choose between options A and B (56 trials) or options C and D (56 trials). The options with the larger maximum outcomes were termed as high-risk (options B and D), and the options with the smaller maximum outcomes were termed as low-risk (options A and C). Positions of options on the computer screen changed across trials in pseudo-random order. At the beginning of the pilot experiment, participants were informed that presented options differed in expected risks, while the expected values were equal for high-risk and low-risk options. According to Brown and Braver (2007), expected risk of each option was defined as [loss probability × (rewards – losses)], expected value of each option was defined as [(reward probability × rewards) + (loss probability × losses)].

**Table 1:** **Reinforcement schedule in the probabilistic two-choice gambling task**.

	**Low-risk options (A)**	**High-risk options (B)**	**Low-risk options (C)**	**High-risk options (D)**
Reward (75%)	+7	+43	+8	+120
Losses (25%)	−9	−117	−12	−348
Expected risk	−0,5	−18,5	−1	−57
Expected value	3	3	3	3

#### Data analyses

To assess risk-taking behavior, percentages (relative to the total amount of choices) of low-risk (options A and C) and high-risk choices (options B and D) were determined and analyzed using two-tailed *t*-tests. Percentages of low-risk and high-risk choices were further analyzed as a function of total account balance (positive account balance; i.e., >0€ vs. negative account balance; i.e., <0€), performing two-tailed *t*-tests. Moreover, we analyzed whether the probability of high-risk choices on a given trial varied as a function of prior feedback valence and prior risk-taking behavior. This was done with an ANOVA with the within-subject factors previous feedback valence (gains vs. losses on the previous trial) and previous risk-taking (low-risk options vs. high-risk options on the previous trial). Statistical analysis was carried out with the Predictive Analytic Software (PASW) 19.0 for Windows.

### Results

Table 2 presents the behavioral results. A significant preference for the low-risk options over the high-risk options was found throughout the task, [*t*_(49)_= 2.84, *p* = 0.007]. The analysis of risk-taking as a function of actual account balance revealed that participants avoided high-risk options when their current balance was positive, [*t*_(49)_= 4.71, *p* < 0.001]. By contrast, high-risk options were preferred when the current balance was negative, [*t*_(49)_= 4.05, *p* < 0.001]. Further, it was shown that risk preference varied as a function of prior feedback valence, [*F*_(1, 49)_= 25.62, *p* < 0.001], and prior risk-taking, [*F*_(1, 49)_= 14.85, *p* < 0.001]. The interaction of feedback valence and prior risk-taking was not significant, [*F*_(1, 49)_ < 1, *p* = 0.970]. These effects reflect that participants preferred higher risks following losses than following gains, as well as following a high-risk decision as compared to a low-risk decision.

**Table 2:** **Behavioral results of the pilot experiment (*N* = 50) and the main experiment (*N* = 20) presenting mean (M) and standard deviation (SD)**.

	**Low-risk options**		**High-risk options**		***T***	**df**	***p***
	**M (%)**	**SD (%)**	**M (%)**	**SD (%)**			
**PILOT EXPERIMENT**							
−	56.9	17.2	43.1	17.2	2.84	49	0.007
Positive balance	49.1	18.1	29.5	13.9	4.71	49	0.000
Negative balance	7.7	8.4	13.6	8.4	−4.05	49	0.000
**MAIN EXPERIMENT**							
−	59.2	19.7	40.8	19.7	2.09	19	0.050
Positive balance	49.0	20.4	26.3	13.5	3.15	19	0.005
Negative balance	10.1	8.3	14.5	8.4	−1.75	19	0.096

Note: Percentages of low-risk and high-risk choices were analyzed as a function of total account balance (positive account balance; i.e., > 0€ vs. negative account balance; i.e., < 0€), performing two-tailed *t*-tests.

### Discussion

With this pilot experiment we aimed to explore risk-taking behavior using a probabilistic two-choice gambling task. During each trial, participants were required to choose between options associated with two different risk levels. As expected, participants preferred the low-risk options over the high-risk options, although options did not differ with respect to expected values. Results are consistent with previous findings of Polezzi et al. (2008). In that study, participants had to choose between a predictable option (which was always associated with a gain of 10€) and an unpredictable option (which was associated with a gain of 30€ or a loss of 10€). The results showed a clear preference for options associated with a predictable outcome, although the expected value of both options was identical. Analysis of the choice history also revealed a loss avoidance tendency among participants. Participants strongly avoided the high-risk options following gains and when they had positive balances. This was not the case after losses and with negative account balances. When faced with rewarding feedback, participants were possibly more willing to protect the money they had and thus showed more conservative behavior. By contrast, the increase in risk proclivity might occur due to an anticipation of larger monetary rewards in order to reduce negative consequences (in terms of corrective actions). These findings are in line with previous studies (Gehring and Willoughby, 2002; Goyer et al., 2008), showing that participants are more likely to engage in risky choices following losses. In summary, the pilot experiment demonstrated the usefulness of the two-choice gambling task as a suitable test for examining risk-taking behavior in unambiguous situations.

## Main experiment

### Materials and methods

#### Participants

20 participants (five men) attended in the ERP study. Their mean age was 29.5 years (SD: 8.9; range, 21–52). All participants were right-handed and had no history of neurological or psychiatric disorders. All participants received verbal and written explanations of the purpose and procedures of the study, and gave written informed consent in accordance with the Declaration of Helsinki. Participants gave written informed consent in accordance with the Declaration of Helsinki.

#### Task and procedure

Task and procedure for Experiment 2 were identical to the pilot experiment except that it comprised of 640 trials that were divided into three blocks with short breaks between blocks. Again, positions of high and low-risk options (left or right on the screen) varied across trials in pseudo-random order. In each trial, participants had to chose between options A and B (320 trials), or options C and D (320 trials). The increase in trial number was necessary to obtain a sufficient number of trials for ERP analyses in all conditions. The experiment lasted about 40 min. All participants were paid 15€ for their participation. To ensure ecological validity of the task and to enhance motivation, participants were informed that they would additionally receive the highest amount they earned in one of the three blocks. The average earning was 6.70€ in this experiment.

#### EEG recording and data analyses

The electroencephalogram (EEG) was recorded from 64 electrodes sites including Cz as a recording reference, using an equidistant electrode system (EASYCAP GmbH, Herrsching-Breitbrunn, Germany). The montage also included additional electrodes that were placed on external locations: below the left and right eye (IO1 and IO2) and in the neck. The ground electrode was located below T1. Electrode impedances were kept below 5 kΩ. Electrical activity was sampled digitally at a rate of 500 Hz, using a time constant of 10 s and a low-pass filter of 250 Hz. Individual electrode positions were digitized based on the run-time measurement of ultrasonic pulses using ELPOS (zebris Medical GmbH, Isny i. Allgäu, Germany). Offline, the EEG data were re-referenced to average reference and corrected for eye-movement artifacts using the multiple source eye correction method as implemented in BESA 5.1 (Brain Electrical Source Analysis, MEGIS Software GmbH, Gräfelfing, Germany). For the FRN and P300 analyses, raw data were filtered offline with a low-pass filter of 40 Hz and an additional notch filter at 50 Hz. Feedback-locked epochs were obtained for each trial, starting 200 ms prior to feedback onset and continuing for 1000 ms post-feedback. Individual averages were baseline-corrected to an average activity between –200 and 0 ms before feedback onset. Feedback-locked epochs were excluded from further analyses if they still contained artifacts. For each participant, ERPs were averaged separately for feedback valance (positive vs. negative) and risk levels (low-risk options vs. high-risk options).

Three components were analyzed. The P200 is a positive component peaking at around 200 ms after feedback onset and measured over frontal areas (Carretié et al., 2001; Polezzi et al., 2008). In the present study, the P200 was determined as the most positive peak between the time window of 100 to 300 ms at electrodes Fz and FCz. FRN amplitudes were computed as the difference between the most negative peak following feedback onset in a 200 to 400 ms time window and the preceding positive peak in the 100 to 300 ms time window at electrodes Fz and FCz. Prior to peak detection, ERPs were filtered with a 15 Hz low-pass filter. The P300 was quantified at CPz and Pz and defined as the mean amplitude in the time range between 300 and 400 ms after feedback presentation. ERP time windows were based on the visual inspection of the grand-average waveforms (for P200 and FRN: Fz, for P300: Pz). Repeated-measurement ANOVAs were computed for the analysis of ERP data, with the within-subject factors electrode (for P200 and FRN: Fz and FCz, for P300: CPz and Pz), feedback valence (gains vs. losses) and risk option (low-risk options vs. high-risk options). Analyses of the behavioral data were identical to the pilot experiment. Correlation coefficients (Pearson *r*) were used to examine associations between FRN and P300 magnitude and percentage of low-risk choices (relative to the percentage of the high-risk choices). All statistical tests were two-tailed.

### Results

#### Behavioral results

As in the pilot experiment, we found a preference for low-risk options over high-risk options, [*t*_(19)_= 2.09, *p* = 0.050] (cf. Table 2). The analysis of risk-taking as a function of account balance revealed that participants avoided high-risk options more when their current balance was positive compared to when it was negative, [*t*_(19)_= 3.15, *p* = 0.005]. Unlike the pilot data, participants only tended to increase risk-taking behavior when their current balance was negative compared to when it was positive, [*t*_(19)_= 1.76, *p* = 0.096]. Furthermore, it was found that the proportion of chosen high-risk options varied as a function of previous outcome valence and previous risk-taking. Consistent with the pilot experiment, we found main effects for feedback valence, [*F*_(1, 19)_= 16.98, *p* = 0.001], and for risk-taking, [*F*_(1, 19)_= 11.57, *p* = 0.003]. Following losses, participants made more risky decisions than after gains. Following risky choices, participants were more likely to choose a high-risk option than after having made a low-risk choice. Finally, a significant interaction was found between previous feedback valence and previous risk-taking, indicating that most high-risk choices were made after high-risk choices followed by negative feedback, [*t*_(19)_= 4.37, *p* < 0.001].

#### ERP results

**Figure 2:**
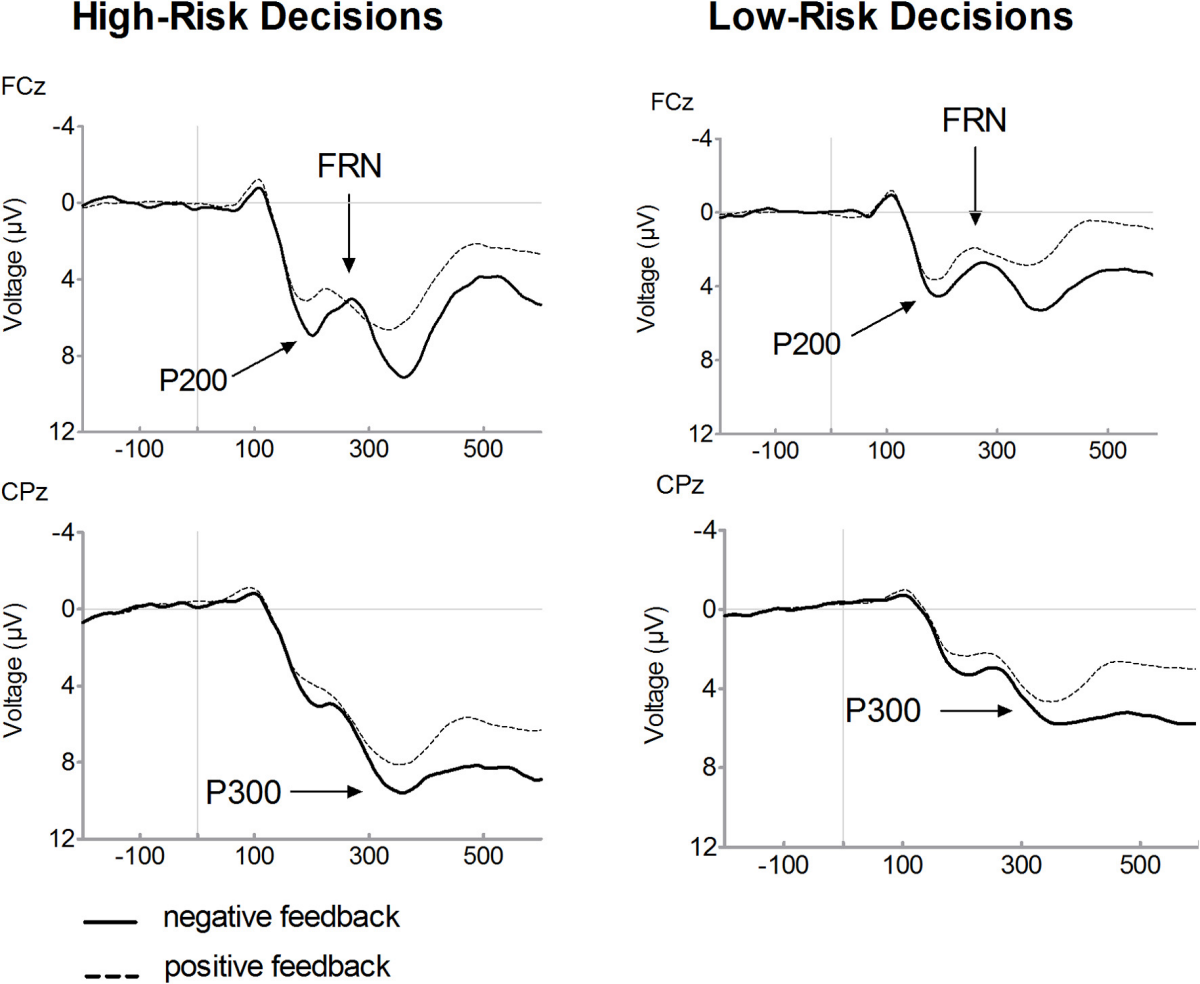
**Feedback-related brain potential waveforms for high-risk and low-risk decisions.** Averaged feedback-locked event-related brain potential (ERP) waveforms are presented for recording sites FCz and CPz. ERPs for high-risk decisions are depicted in the left columns and for low-risk decisions in the right column. Waveforms for negative feedback (solid line) and for positive feedback (dashed line) are overlaid. The P200 was determined as the most positive peak between 100 and 300 ms. The FRN was computed as the difference between the most negative peak following feedback onset in a 200 to 400 ms time window and the preceding positive peak between 100 and 300 ms. The P300 was defined as the mean amplitude in the time range between 300 and 400 ms after feedback presentation.

**Figure 3:**
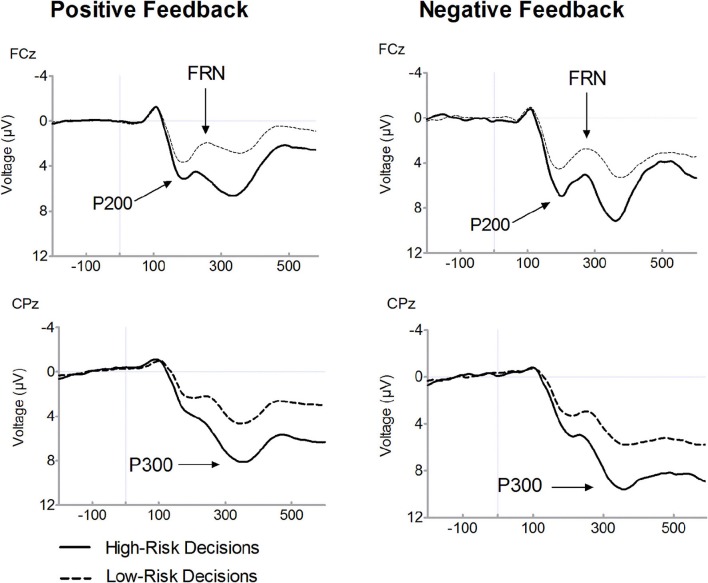
**Feedback-related brain potential waveforms for positive (i.e., gains) and negative feedback (i.e., losses).** Averaged feedback-locked event-related brain potential (ERP) waveforms are presented for recording sites FCz and CPz. ERPs for positive feedback (i.e., gains) are depicted in the left columns and for negative feedback (i.e., losses) in the right column. Waveforms for high-risk decisions (solid line) and for low-risk-decisions (dashed line) are overlaid.

Figure 2 presents feedback-locked ERP waveforms on positive (dashed line) and on negative (solid line) feedback trials, separately for selected high-risk and low-risk options. Figure 3 displays ERPs for positive and negative feedback trials for a comparison of the high-risk (solid line) and low-risk options (dashed line). Inspection of ERPs indicated three distinct components related to risk processing. The first component is the P200, an early positive wave peaking at a latency of approximately 200 ms. The FRN is the negative deflection between two positive components. The third component is the P300, peaking approximately between 300 and 400 ms following feedback onset. Losses elicited large P300 amplitudes which may overlap with the FRN effect. It is noteworthy that ERP waveforms indicate that FRN peak amplitudes were lower for losses than for gains which are mainly due to the variation of P200 and P300 amplitudes. Therefore, FRN amplitudes were calculated as the difference between the most negative peak amplitude minus the preceding positive peak amplitude. With regard the P200, a significant main effect of valence was found, [*F*_(1, 19)_= 67.56, *p* < 0.001], indicating larger amplitudes for losses compared to gains. There was also a significant main effect of risk, [*F*_(1, 19)_= 43.87, *p* < 0.001], revealing enhanced amplitudes in high-risk options compared to low-risk options. Furthermore, a significant interaction of risk and valence was found, [*F*_(1, 19)_= 19.36, *p* < 0.001]. In high-risk decisions, larger amplitude differences between positive and negative feedbacks were found which was due to enhanced P200 amplitudes on negative feedbacks in high-risk options. The main effect of electrode was also significant, [*F*_(1, 19)_= 34.59, *p* < 0.001], with larger P200 at FCz compared to Fz.

Consistent with previous findings, a main effect of valence was found for FRN amplitudes, indicating enhanced FRN amplitudes on losses compared to gains, [*F*_(1, 19)_= 11.08, *p* = 0.004]. Furthermore, the FRN was modulated by risk as evidenced by a significant interaction between valence and risk option, [*F*_(1, 19)_= 10.12, *p* = 0.005]. After choosing the high risk option, large amplitude differences between losses and gains were found, [*t*_(19)_= 4.03, *p* = 0.001]. By contrast, there was no significant amplitude difference between losses and gains in the low-risk condition, [*t*_(19)_= 1.08, *p* = 0.293]. While the FRN on losses did not differ with respect to the riskiness of options, [*t*_(19)_ = 0.65, *p* = 0.522], amplitudes on gains were reduced in the high-risk compared to the low-risk option, [*t*_(19)_ = −3.55, *p* = 0.002]. The main effect of electrode also approached significance, [*F*_(1, 19)_= 3.46, *p* = 0.079], due to larger FRN amplitudes at Fz relative to FCz.

The P300 is also depicted in Figures 2 and 3. A main effect of valence was found, [*F*_(1, 19)_= 18.06, *p* < 0.001], indicating that the P300 was larger on losses compared to gains. There was a significant main effect of risk option, [*F*_(1, 19)_= 36.08, *p* < 0.001], showing enhanced amplitudes in high-risk compared to low-risk choices. No significant interaction between feedback valence and risk option was observed (*F* < 1). The P300 tended to be larger at CPz compared to Pz, [*F*_(1, 19)_= 3.48, *p* = 0.078].

#### Correlational findings

Bivariate correlations were computed relating ERPs following losses and following gains (averaged across FCz and Fz for FRN analyses, and across CPz and Pz for P300 analyses) to the percentage of low-risk choices. Negative correlations for FRN amplitudes indicate an increase in FRN (i.e., more negative amplitudes) with risk aversion.

We found significant correlations between risk avoidance and FRN amplitudes on gains (*r* = −0.47, *p* = 0.039), the FRN on losses at a trend level (*r* = −0.41, *p* = 0.073), and P300 amplitudes on losses (*r* = 0.52, *p* = 0.018). No significant correlations were found between risk-avoidance and P300 amplitudes after gains. Note that FRN and P300 amplitudes were not correlated (*r* = −0.02, *p* = 0.91).

## General discussion

The current study focused on decision-making and its neural correlates using a monetarily motivated probabilistic two-choice gambling task. In accordance with previous findings, participants were generally risk-averse but modulated their risk-taking behavior according to reward history (Tversky and Kahneman, 1981). ERP research on decision-making has left the question unanswered as to how electrophysiological indicators are specifically affected by different risk levels. In order to address this issue, we independently controlled for different risk parameters.

Differential processing of risky decisions was reflected in the FRN amplitudes. The FRN was modulated by the well-known distinction between gains and losses (Gehring and Willoughby, 2002; Hajcak et al., 2006), but only in high-risk options. Considered in the framework of the RL theory of the FRN (Holroyd and Coles, 2002), it appears that the reward prediction error signal is increased after high-risk decisions, where larger potential positive and negative consequences were expected. The null effect under the low-risk condition might be explained by generally smaller reward prediction errors generated in the low-risk condition, characterized by small positive and small negative outcomes. Results are also in line with Holroyd et al. (2004), showing that the FRN reflects loss sizes in relation to what was expected. Whereas Holroyd and Coles (2002) interpreted the FRN purely as a reinforcement signal, Gehring and Willoughby (2002) suggested that the FRN might also reflect the motivational impact of ongoing events. Thus, modulations in high-risk decisions may also reflect the motivational or emotional significance of high-risk decisions compared with low-risk decisions. Due to the potential negative consequences of high-risk choices, discriminating between losses and gains seems highly important for optimizing future decision-making. Consistent with this interpretation, brain imaging studies emphasized the role of the ACC in evaluating unfavorable outcomes (Bush et al., 2000; Luu et al., 2000) and in risk assessment (Ernst et al., 2004; Fukui et al., 2005). Also, cingulate lesions in monkeys have been shown to impair the ability to use previous reinforcements to guide future decision-making behavior (McCoy and Platt, 2005; Kennerley et al., 2006). Alternatively, the FRN pattern in high-risk decisions might be due to variations of the P200 which was also affected by outcome valence. However, the P200 was enhanced on negative feedback trials in high-risk options whereas the FRN on negative feedbacks did not vary with risk. Interestingly, the interaction of feedback valence and risk was mainly caused by FRN amplitude variations on positive feedbacks. In the present study, reduced amplitudes were found for positive feedbacks in high-risk relative to low-risk choices. Larger amplitudes were found for lower outcomes and for smaller positive reward prediction errors. Our results contribute to a growing debate about the relevance of positive feedback for reward-related processes and several studies found greater modulation of amplitudes on gain trials compared to loss trials (Cohen et al., 2007; Holroyd et al., 2008). It has been shown that the amplitude following positive feedbacks varied with the probability of reward. Thus it was argued that it may represent the magnitude of the positive reward prediction error (Cohen et al., 2007). Possibly, the current result of reduced FRN amplitudes in the high-risk condition may be a consequence of the larger positive reward prediction error associated with gains in that condition. However, few studies examined the effects on positive feedbacks, and consistent patterns of results have not been observed yet. Therefore, these results have to be interpreted with caution, and further studies are needed to examine the effects of reward prediction error on different risk parameters.

Decision riskiness also affected the P200, a component that has previously been associated with the attention processing of emotional stimuli, such as faces. The more negative the valence, the larger the P200 amplitude (Carretié et al., 2001, 2005). Consistent with these findings, in the present study negative feedback (i.e., losses) and high-risk decisions induced larger P200s. The present results indicate that amplitudes were modulated by outcome magnitude, especially in high-risk gambles. Polezzi et al. (2008) reported that unpredictable outcomes are associated with larger P200 amplitudes compared to predictable outcomes, which is consistent with the current results. But, in that study the P200 was not sensitive to the distinction between positive and negative outcome. Our data suggest an early processing of negative feedback and of high-risk decisions. Also, Bellebaum et al. (2010) revealed that P200 amplitudes are larger under a reward outcome condition compared to a non-reward outcome condition both in active and observational gambling tasks. Moreover, the active execution induced a larger discrepancy of P200 amplitudes between reward and non-reward than that of passive observation. The present findings suggest an enhanced sensitivity in risky decisions to the gain- and loss-outcome difference at a very early stage. Importantly, we found large reward prediction errors in high-risk options as early as in the P200 time range. Possibly, the P200 codes the most relevant features of a context, especially when risky decisions have to be made in order to avoid future negative consequences.

In agreement with previous studies (Frank et al., 2005; Schuermann et al., 2011), P300 amplitudes were enhanced on negative compared to positive feedbacks. In addition to valence effects, enhanced P300 amplitudes were found in high-risk options relative to low-risk options. Both results are in accordance with the finding that the feedback-related P300 is sensitive to reward probability (Bellebaum and Daum, 2008; Pfabigan et al., 2011) since negative outcomes were less probable in the current study and with the finding that the P300 is sensitive to reward magnitude since negative outcomes in the high-risk condition were of greater magnitude (Yeung and Sanfey, 2004; Hajcak et al., 2005, 2007). The P300 enhancement in high-risk choices may reflect enhanced motivational significance of risky decisions. This is supported by the hypothesis that the P300 may reflect motivational processes linked to noradrenergic transmission (Nieuwenhuis et al., 2005).

Interestingly, FRN and P300 appear to reflect risk-taking behavior, but they might reflect different aspects of risky decision-making. Whereas FRN amplitudes are reduced by large positive prediction errors, P300 amplitudes are enhanced due to larger negative outcomes. Importantly, risk-avoidance behavior was associated with enhanced FRN and P300 amplitudes. Possibly, increased FRN amplitudes reflect enhanced cognitive control that is essential for the avoidance of risky decisions. The association between FRN and risk-avoidance is in line with previous findings, revealing an inverse relation between ERN/FRN amplitudes and risk-taking behavior in healthy individuals (e.g., Hewig et al., 2007; Santesso and Segalowitz, 2009) and in patients with borderline personality disorder (Ruchsow et al., 2006; Schuermann et al., 2011). FRN findings complement brain imaging results, suggesting that greater ACC activity predicts less risk-seeking behavior (Paulus and Frank, 2006). Nonetheless, interpretation of correlation analyses of the present study should be cautiously interpreted due to the relatively small sample size.

To our knowledge, this is the first study that independently controlled for different risk parameters. Nevertheless, there are possible confounds that should be discussed. First, the comparison between positive and negative feedbacks is confounded with feedback probability, i.e., positive feedbacks were more frequent than negative feedbacks. Therefore, reward probability may have influenced the difference between positive and negative feedbacks (Holroyd et al., 2003; but: Hajcak et al., 2005). While this should have affected the distinction between positive and negative feedbacks in both conditions, an amplitude difference was only found in the high-risk condition. In addition, feedback probability should not affect the comparison of feedback types between high- and low-risk gambles, since respective feedback probabilities were equal in both conditions. Second, although we aimed to disentangle expected risk from feedback probability, we could not independently manipulate outcome magnitude and reward prediction errors. Future studies on decision-making behavior under risk should further examine the influence of valence, magnitude, and expected risk on behavioral and ERP parameters to describe the underlying neural mechanisms more precisely. In particular, future research could parametrically vary risk parameters such that they vary from trial to trial in a decorrelated fashion.

To conclude, the present findings indicate that the processes underlying human decision-making are significantly affected by decision riskiness when controlling for reward probility and expected value. The increased P200 on negative feedback in high-risk decisions suggests that large reward prediction errors are processed as early as in the P200 time range. The FRN is affected by feedback valence depending on decision riskiness. Considered in the framework of the RL theory of the FRN, it has been suggested that the reward prediction error signal is increased after high-risk decisions compared to low-risk decisions. The later P300 amplitude is sensitive to feedback valence as well as to the risk associated with a decision. Thus, the P300 carries additional information for reward processing, mainly the enhanced motivational significance of risky decisions. Due to the potential negative consequences of high-risk choices, rapidly processing the relevant and informative features of a context when decisions have to be made seemed highly important for optimizing future decision-making. Risk-taking is a central cognitive-motivational construct accounting for many everyday decisions. In addition, understanding the neurocognitive basis of risk-taking behavior might also be central to explaining certain symptoms of psychopathological conditions, e.g., borderline personality disorder, patients with bipolar disorders or patients with substance dependency.

### Conflict of interest statement

The authors declare that the research was conducted in the absence of any commercial or financial relationships that could be construed as a potential conflict of interest.
